# Molecular profiling of advanced breast cancer tumors is beneficial in assisting clinical treatment plans

**DOI:** 10.18632/oncotarget.24564

**Published:** 2018-02-24

**Authors:** Philip Carter, Costi Alifrangis, Biancastella Cereser, Pramodh Chandrasinghe, Lisa Del Bel Belluz, Nina Moderau, Fotini Poyia, Lee S. Schwartzberg, Neha Tabassum, Jinrui Wen, Jonathan Krell, Justin Stebbing

**Affiliations:** ^1^ Department of Surgery and Cancer, Imperial College, London, UK; ^2^ Department of Oncology, University College Hospital, London, UK; ^3^ Department of Surgery, University of Kelaniya, Kelaniya, Sri Lanka; ^4^ West Cancer Center, The University of Tennessee, Memphis, USA

**Keywords:** tumor profiling, breast cancer, cancer treatment

## Abstract

We used data obtained by Caris Life Sciences, to evaluate the benefits of tailoring treatments for a breast carcinoma cohort by using tumor molecular profiles to inform decisions. Data for 92 breast cancer patients from the commercial Caris Molecular Intelligence database was retrospectively divided into two groups, so that the first always followed treatment recommendations, whereas in the second group all patients received at least one drug after profiling that was predicted to lack benefit. The biomarker and drug associations were based on tests including fluorescent *in situ* hybridization and DNA sequencing, although immunohistochemistry was the main test used.

Patients whose drugs matched those recommended according to their tumor profile had an average overall survival of 667 days, compared to 510 days for patients that did not (P=0.0316). In the matched treatment group, 26% of patients were deceased by the last time of monitoring, whereas this was 41% in the unmatched group (P=0.1257). We therefore confirm the ability of tumor molecular profiling to improve survival of breast cancer patients. Immunohistochemistry biomarkers for the androgen, estrogen and progesterone receptors were found to be prognostic for survival.

## INTRODUCTION

Breast cancer is the most prevalent form of cancer in women, causing approximately one in four of all cases worldwide. In 2012, there were 1.68 million diagnoses and 522,000 deaths according to the World Health Organization, and around 80% of cases occur within patients over the age of 50. Risk factors include obesity, lack of exercise, alcohol consumption, age, family history and age at menarche. The long-term outcome for patients depends on the stage of the tumor and its characteristics at diagnosis.

Established evidence-based treatments for advanced disease includes radiation, chemotherapy, hormonal therapy, and targeted therapies. Due to the application of these treatments, in the developed world survival is relatively high, with between 80% and 90% of those in England and the USA surviving for at least five years.

Hereditary genetic factors are thought to play a minor role in sporadic breast carcinoma, but in approximately 5% of cases it is significant. Germline mutations in the genes *BRCA1*, *BRCA2*, *p53*, *PTEN*, *STK11*, *CHEK2*, *ATM*, *BRIP1* and *PALB2* are all considered important in breast cancer tumorigenesis. The genetics of sporadic breast cancer is now better understood, due to genomic sequencing of many such tumors [1]. Somatic driver variants and the mutational processes underlying them have now been identified [2], and the sequencing of 560 breast cancer genomes [3] has furthered progression towards a complete description of the molecular events that cause these tumors. In total, 93 protein-coding cancer genes were found to have probable driver mutations. This and other data including exon sequencing [4], whole genome [5], transcriptional [6], and methylation-based studies [7] have been used to develop a molecular taxonomy of breast cancer.

Molecularly defined characteristics have been used as predictive and prognostic biomarkers in breast cancer to define therapeutic approaches. The earliest such example is the identification of the estrogen receptor (ER) overexpression in a subset of breast carcinomas in the 1970s, and its subsequent targeting with ER directed therapies [8]. Similarly *HER2* (human epidermal growth factor receptor 2) overexpression and its targeting with Herceptin [9] have further defined a subset of this disease that behaves and responds uniquely to HER2-directed therapies. Gene chip technologies that use gene expression profiling of the primary tumor such as OncotypeDX, have been FDA approved as a decision aid in early breast cancer to help define prognostic features [10]. Several preclinical studies have identified drug-genome interactions [11]. These have been borne through with great successes in specific situations, such as the *EML4-ALK* translocation in non-small cell carcinoma of the lung. These specific tissue and gene scenarios have been validated in prospective clinical studies, and have transformed clinical practice [12].

An approach that has gained traction in recent years is the application of molecular characterization beyond established immunohistochemistry (IHC) biomarkers. This has been used to guide therapeutic decision making across many tissue types, after failure of standard therapies. Genomic sequencing of the cancer [13] enables identification of somatic driver variants, which have been associated with therapeutic outcomes in preclinical or clinical studies [14]. The efficacy of this approach is currently unclear across tumor types, as some mutations are only known to be prognostic for response in particular situations; some early attempts at matching therapies failed for this reason, e.g. the use of BRAF inhibitors in *BRAF* mutant colorectal cancer [15].

It has been shown that tumor profiling of non-responsive breast cancer resulted in better clinical treatments [14], while other studies have demonstrated the benefit of profiling in other tumor types [16]. To investigate the effectiveness of one such profiling method, we evaluated data provided by Caris Life Sciences from their CODE database (version 1.0). This resource describes molecular profiling data that has been used to recommend clinical treatments, and drug regimens used before and after clinicians received this information along with their outcomes. The impact of profiling on drug usage and survival was evaluated here.

## RESULTS

### Patient characteristics

Data from the Caris CODE database of 92 advanced stage breast cancer patients who underwent treatment was analyzed. These patients were retrospectively divided into two groups, based on their matching of treatments to recommendations that had been generated according to their profiles. In the matched treatment group, 43 patients received at least one recommended drug after collection of tumor sample for profiling and none that were predicted to lack benefit, whereas in the unmatched treatment group 49 patients were given one or more drugs that were classified as having a lack of benefit at any time following profiling. Information about the patients in both groups is summarized in Table 1 (age, ethnicity, histology, tumor grade and stage, and survival information).

**Table 1:** A summary of patient information comparing the matched and unmatched groups against all patients overall

Patient & Tumor Information							
Group	Age	Ethnicity	Histology	Grade	Stage	Survival (Days)	Mortality
All patients (92)	57	White: 71 Black/African American: 13 Asian: 4 Hawaiian/Pacific Islander: 2 American Indian/Alaskan Native: 1 Other/Unknown: 1	Infiltrating duct carcinoma, NOS: 50 Infiltrating ductular carcinoma: 11 Carcinoma, NOS: 10 Lobular carcinoma, NOS: 5 Adenocarcinoma, NOS: 4 Infiltrating lobular carcinoma, NOS: 3 Metaplastic carcinoma, NOS: 2 Ductal carcinoma, NOS: 2 Intraductal papillary adenocarcinoma with invasion: 1 Infiltrating duct and lobular carcinoma: 1 Infiltrating duct mixed with other types of carcinoma, *in situ*: 1 Intraductal papillary-mucinous carcinoma, invasive: 1 Infiltrating lobular mixed with other types of carcinoma: 1	Grade 3/ Poorly differentiated: 41 (45%) Grade 2 / Moderately differentiated: 44 (48%) Grade 1 / Well differentiated: 2 (2%) Unknown / Not determined: 4 (4%) None / Not applicable: 1 (1%)	IV: 18 (19%) III no IIIC: 21 (23%) IIIC: 9 (10%) II: 31 (34%) I: 10 (11%) Unknown: 3 (3%)	583	34%
Matched only (43)	55.8	White: 34 Asian: 3 Black/African American: 3 American Indian/Alaskan Native: 1 Other/Unknown: 1 Hawaiian/Pacific Islander: 1	Infiltrating duct carcinoma, NOS: 21 Carcinoma, NOS: 8 Infiltrating ductular carcinoma: 5 Adenocarcinoma, NOS: 2 Infiltrating lobular carcinoma, NOS: 2 Lobular carcinoma, NOS: 2 Ductal carcinoma, NOS: 1 Metaplastic carcinoma, NOS: 1 Infiltrating duct and lobular carcinoma: 1	Grade 3/ Poorly differentiated: 15 (35%) Grade 2 / Moderately differentiated: 28 (65%)	II: 13 (30%) III no IIIC: 10 (23%) IV: 10 (23%) IIIC: 4 (10%) I: 3 (7%) Unknown: 3 (7%)	667	26%
Unmatched (49)	58.1	White: 37 Black/African American: 10 Asian: 1 Hawaiian/Pacific Islander: 1	Infiltrating duct carcinoma, NOS: 29 Infiltrating ductular carcinoma: 6 Lobular carcinoma, NOS: 3 Adenocarcinoma, NOS: 2 Carcinoma, NOS: 2 Ductal carcinoma, NOS: 1 Infiltrating lobular carcinoma, NOS: 1 Infiltrating duct mixed with other types of carcinoma, *in situ*: 1 Intraductal papillary adenocarcinoma with invasion: 1 Metaplastic carcinoma, NOS: 1 Infiltrating lobular mixed with other types of carcinoma: 1 Intraductal papillary-mucinous carcinoma, invasive: 1	Grade 3/ Poorly differentiated: 26 (53%) Grade 2 / Moderately differentiated: 16 (33%) Grade 1 / Well differentiated: 2 (4%) Unknown / Not determined: 4 (8%) None / Not applicable: 1 (2%)	IV: 8 (16%) III no IIIC: 11 (23%) IIIC: 5 (10%) II: 18 (37%) I: 7 (14%)	510	41%

### Treatment analysis

**Figure 1:**
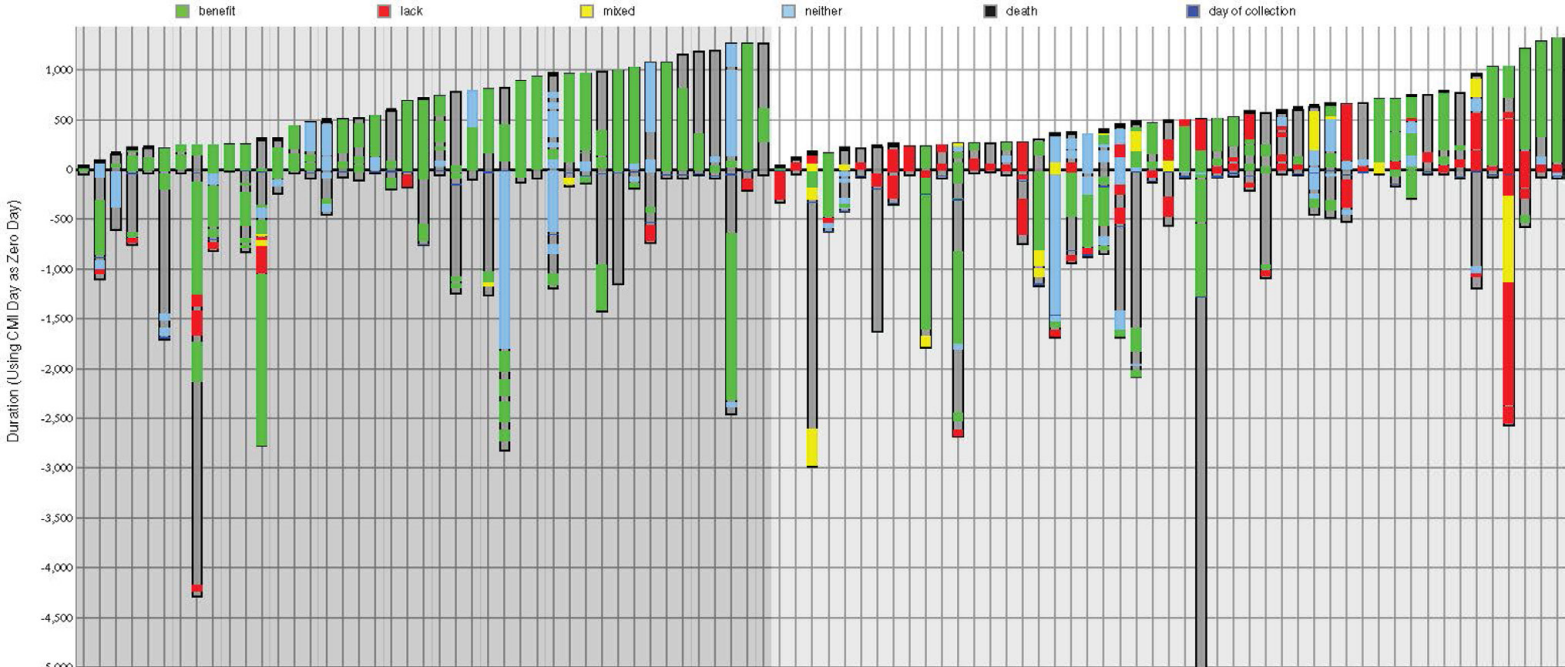
Treatments ordered by survival time for matched and unmatched patients On the left (darker gray background) - treatment regimens followed by 43 matched patients, in ascending post-profiling survival time; on the right (lighter gray background) - 49 unmatched patients ordered by post-profiling survival time. Each column represents one patient. The y-axis is time (days) where zero is the time of profiling. Dark gray within a column shows the total time monitored from diagnosis to either death or last follow-up; a black line at the top of a column indicates death; green bars represents time on a drug of benefit; red is a lack of benefit drug; yellow is time on a combination therapy associated with both benefit and lack of benefit. Blue bars represent time on a neutral therapy associated with neither benefit nor lack of benefit.

Do patients whose treatments consistently follow profile-based recommendations fare better than patients whose treatments that do not? To compare the overall survival of the two groups that will be referred to as matched and unmatched, waterfall plots for both are shown in Figure 1, where each bar represents a treatment schedule for a breast cancer patient. The 92 bars shown denote 43 matched and 49 unmatched patients (on the left and right respectively). Each set is ordered by survival time following profiling, so that from left to right in the plots patients are displayed as their post-profiling survival time increases. Green lines indicate administration of drugs predicted to be of benefit (and therefore more prevalent in the matched group), red lines are drugs that have a lack of benefit, and yellow corresponds to times when both of these types of drug were received by the patient. The recommendations from Caris are mostly based on the literature.

Table 2 shows the drugs most frequently given to all patients compared to the matched and unmatched groups. The number of patients treated with a drug is shown in the first column, and the number of continuous treatment periods is shown in all other columns i.e. treatments of the same patient with intervening periods are counted separately. The drugs given to the most number of patients were cyclophosphamide (70 patients), doxorubicin hydrochloride (58) and docetaxel (56). Overall the most commonly administered drugs were cyclophosphamide (given for 76 time periods), doxorubicin hydrochloride (61), and docetaxel (58). In the matched group docetaxel was given more often than doxorubicin hydrochloride, although cyclophosphamide was still given most often. However, in the matched group after profiling, cyclophosphamide, doxorubicin hydrochloride, paclitaxel and trastuzumab were given less frequently.

**Table 2:** Most frequently given drug treatments in the matched and unmatched groups, compared with all patients, and the most popular drugs overall that were predicted to be of benefit, lacking benefit, or neither of these

Number of Patients Treated	Most Frequently Administered Drugs (Total Treatment Periods)							
All Patients Treated	All Patients – Treatment Periods	Matched Only Patients, All Treatments	Matched, After Profiling Treatments Only	Unmatched Patients, All Treatments	Unmatched, After Profiling Treatments Only	Drugs Predicted of Benefit	Drugs Predicted to Lack Benefit	Drugs with No Prediction (Neither of Benefit or Lack of Benefit)
cyclophosphamide – 70 patients	cyclophosphamide (76)	cyclophosphamide (32)	letrozole; docetaxel (11)	cyclophosphamide (44)	docetaxel (13)	letrozole (28)	doxorubicin hydrochloride (32)	cyclophosphamide (73)
doxorubicin hydrochloride – 58 patients	doxorubicin hydrochloride (61)	docetaxel (29)	-	doxorubicin hydrochloride (36)	letrozole (11)	doxorubicin hydrochloride; trastuzumab (22)	trastuzumab; docetaxel (11)	docetaxel (24)
docetaxel – 56 patients	docetaxel (58)	doxorubicin hydrochloride (25)	carboplatin; capecitabine (7)	docetaxel (29)	gemcitabine hydrochloride (10)	-	-	paclitaxel (18)
carboplatin – 32 patients	carboplatin (36)	carboplatin (20)	-	trastuzumab (22)	capecitabine (9)	docetaxel (21)	carboplatin (10)	capecitabine (13)
letrozole – 31 patients	trastuzumab (35)	paclitaxel (17)	exemestane; gemcitabine hydrochloride (6)	letrozole (17)	anastrozole (8)	tamoxifen citrate (18)	capecitabine (6)	carboplatin (12)
paclitaxel – 29 patients	letrozole; paclitaxel (32)	letrozole (15)	-	capecitabine; carboplatin; gemcitabine hydrochloride (16)	cyclophosphamide (6)	anastrozole (17)	gemcitabine hydrochloride (5)	gemcitabine hydrochloride (9)
capecitabine – 27 patients	-	trastuzumab (13)	nab-paclitaxel (5)	-	methotrexate; doxorubicin hydrochloride (5)	carboplatin (13)	methotrexate (5)	fulvestrant (8)
gemcitabine hydrochloride; trastuzumab – 22 patients	capecitabine; gemcitabine hydrochloride (27)	gemcitabine hydrochloride; capecitabine; nab-paclitaxel (11)	cyclophosphamide; tamoxifen citrate; anastrozole (4)	-	-	gemcitabine hydrochloride (12)	nab-paclitaxel (4)	nab-paclitaxel (8)
-	-	-	-	paclitaxel (15)	carboplatin; fluorouracil; vinorelbine tartrate (4)	exemestane (9)	anastrozole; pegylated liposomal doxorubicin hydrochloride; paclitaxel (3)	bevacizumab; vinorelbine tartrate (7)
anastrozole; nab-paclitaxel; tamoxifen citrate – 19 patients	anastrozole; nab-paclitaxel (21)	-	-	anastrozole (11)	-	fluorouracil (8)	-	-

Most commonly given drugs are listed in descending order going down, with the total number of treatments shown in parentheses (except the first column, which shows the number of patients treated).

On average patients received 5.8 drug treatments. Of these, 40% (2.3 drugs) were predicted to be of benefit, 19% (1.1 drugs) lacked benefit, and 41% (2.4) being neither. Matched patients on average had 5.6 drug treatments – 49% (2.7 drugs) of these were profiled to be of benefit, 6% (0.4) lacked benefit, and 45% (2.5) being neither. Unmatched patients received an average of 5.9 drug treatments; 32% (1.9 drugs) of these were of benefit, 31% (1.8) lacked benefit, and 37% (2.2) neither.

In the unmatched set, 76% of patients received at least one drug treatment predicted to be of benefit, and 49% received two or more drug treatments of this type.

The most commonly given drugs of benefit were letrozole (28), doxorubicin hydrochloride (22), trastuzumab (22), and docetaxel (21). The most commonly given lack of benefit drugs were doxorubicin hydrochloride (32), trastuzumab (11) and docetaxel (11). Some of the administered drugs did not have a recommendation for or against, and appear in the “neither” category. This neither class makes up 45% of drugs administered in the matched cohort versus 38% in the unmatched cohort. The most common agent by far in the neither category was cyclophosphamide (given for 73 time periods, i.e. 13% of all drug treatments for this cohort).

As might be expected, some of the drugs that were most commonly used were administered at similar rates whether or not they were predicted to be of benefit to the patient. However, 31% of the times that trastuzumab was given it was expected to lack benefit. Doxorubicin hydrochloride was only given 38% of the time when it was thought to be beneficial, while 55% of the time it was predicted to lack benefit. Letrozole was used when profiled to be of help 88% of the time. We note that palbociclib was not used in combination with letrozole in any of the cases in the breast cohort studied here, but most of these cases were prior to the FDA approval of this drug. Tamoxifen citrate was prescribed 95% of the time when it was expected to be favorable, and anastrozole was also well matched, being given 85% of the time that it was predicted to be of value.

### Survival analysis

Patients in the matched group on average survived for 667 days after the day of profiling, compared to 510 days for patients whose treatments did not match their molecular profile (P=0.0316); this is an increase of 31%.

In the matched group 26% of patients were deceased by the end of the time of monitoring compared to 41% of the unmatched group of patients (P=0.1257).

Patients who received more than one drug in the lack-of-benefit category trended towards worse overall survival (OS) than patients who received only a single drug in this category: 550 days versus 461 days.

**Figure 2:**
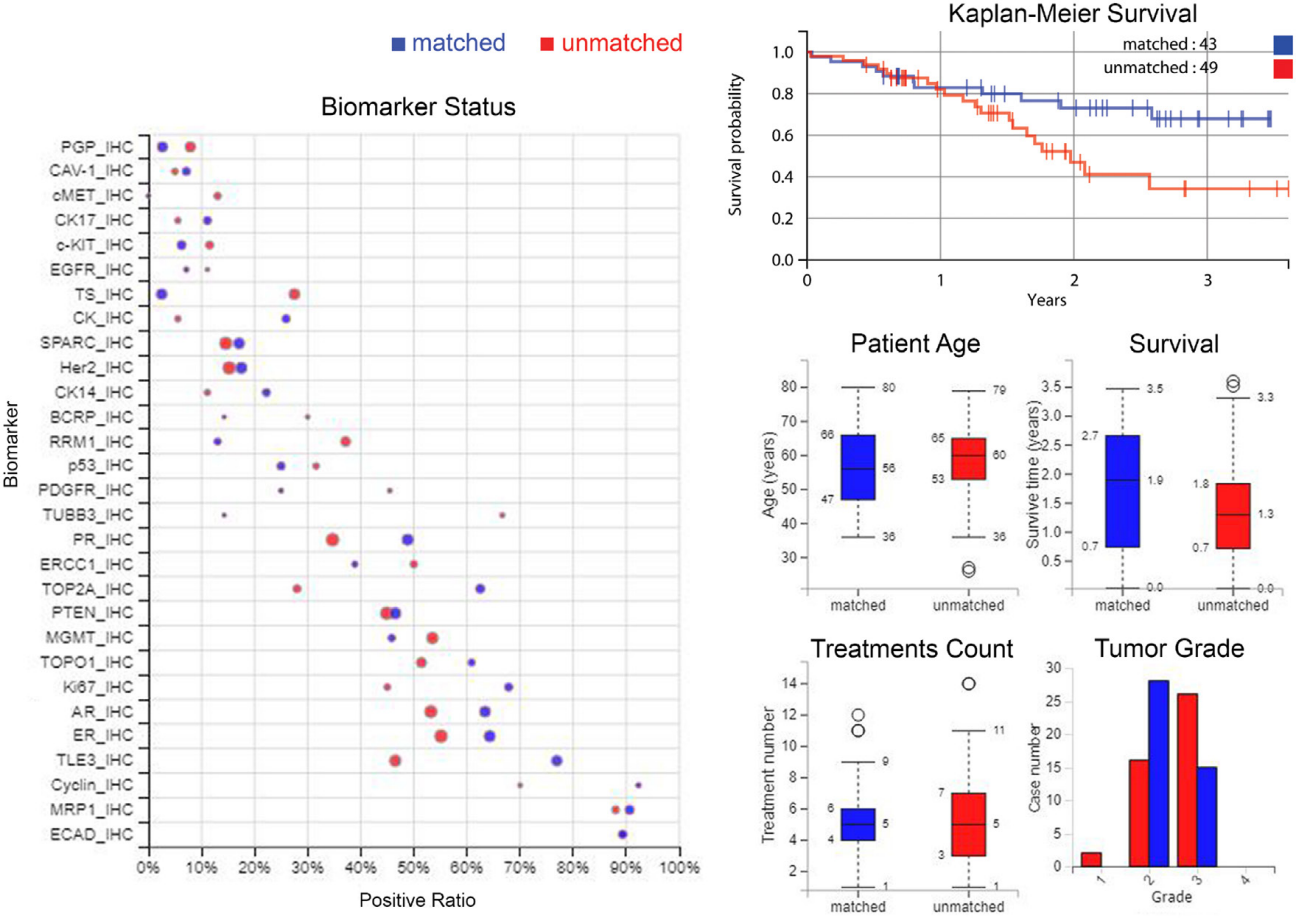
Differences between matched and unmatched groups in biomarker statuses, survival, demographics and tumour grade *Left:* Comparison of biomarkers between matched and unmatched groups; positive ratio represents the percentage of the cases that have “positive” biomarker results. Specifically, for IHC, positive is defined as protein expression being above a predetermined threshold. For sequencing biomarkers, positive is defined as a gene mutation (usually pathogenic). The size of the circle indicates the number of cases. *Top-right*: A Kaplan-Meier curve showing the increase in overall survival from time of profiling for those patients treated only with therapies predicted to be of benefit by their molecular profile, compared to those patients who received at least one therapy predicted to lack benefit. *Middle-right and lower-right*: Comparison of age of patients, survival time, treatment numbers, grade of samples, between matched and unmatched. Blue denotes matched patients and red is unmatched patients in all plots.

A Kaplan-Meier curve (Figure 2, top-right) shows the improvement in OS from time of profiling for patients treated only with therapies predicted to be of benefit by their molecular profile, and separately, patients who received at least one drug predicted to lack benefit. Figure 2 (left) also gives a comparison of biomarkers between matched and unmatched groups, and (middle-right and lower-right plots) matched versus unmatched: age of patients, survival time, treatment numbers and grade of samples.

## DISCUSSION

### Predictive biomarkers – matched treatments better than unmatched

This report looked at data from a breast carcinoma cohort made available from Caris Life Sciences via their CODE database. This was a retrospective review of a cohort of patients that were profiled using established IHC biomarkers, along with fragment analysis, *in situ* hybridization and sequencing. Their treatments were either matched or unmatched based on whether the treatment chosen by their physician was predicted to be beneficial by Caris Life Sciences using the molecular profile of the tumor. Patients whose treatments subsequently agreed with these recommendations were compared to those who received at least one drug that was predicted to lack benefit, i.e. their regimen did not agree with their tumor profile-based treatment predictions. Comparing these two groups showed that the matched treatment group had an increase of 31% in survival compared to the average for the unmatched group, an increase of 157 days from 510 to 667 days (P=0.0316).

When comparing the matched and unmatched groups, in terms of HER2 and ER directed therapies, there was a similar level of use – in the matched group 87% of treatments were of either of these types, and in the unmatched group 83% of the treatments were one of these two types.

The unmatched group received 0.32 more lines of therapy on average than the matched group, survived for less time, and had a higher mortality rate. This could have been influenced by the tendency for the unmatched group to have tumors that were generally more advanced than in the matched group, as shown in Table 1. The unmatched group may have received more treatments overall and been less adherent to the recommended treatments, due to clinicians trying all possible options as a last resort as the disease advanced, although this is speculative.

**Figure 3:**
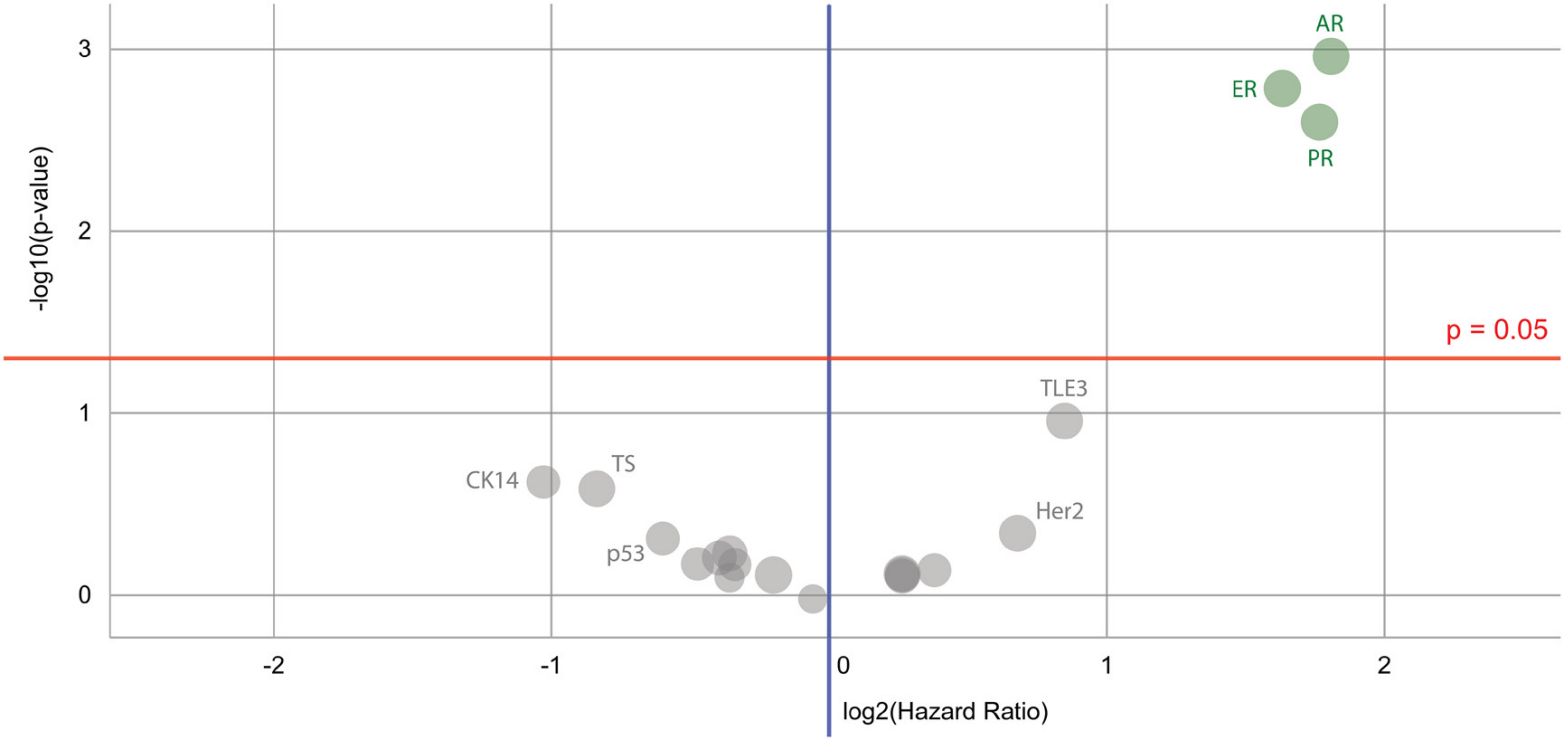
Volcano plot of biomarkers’ prognostic value for a Caris breast cancer dataset Biomarkers of significance that can be used to indicate differences in survival are found in a cluster on the top right – these are the immunohistochemistry androgen receptor (AR), estrogen receptor (ER) and progesterone receptor (PR) markers. Color code: green = the hazard rate of a positive biomarker result is significantly lower than that of a negative biomarker result; gray = the difference between a positive biomarker result and a negative biomarker result is not significant.

Interestingly, across this cohort of patients, the expression of ER and PR was found to be prognostic for overall survival (see Figure 3). The expression of the androgen receptor (AR) was also found to be prognostic; this agrees with previously published data that shows improved long-term survival with co-expression of AR in ER positive breast cancers [16].

The survival curves from time of diagnosis initially overlap and then diverge after profiling occurs. This may suggest that basing therapy on tumor profiling has an effect on selecting optimal therapies and improving outcome. Combined with the increase in survival and lowering of death rates, this leads to the conclusion that there is a beneficent role of tumor molecular profiling in this cohort.

## MATERIALS AND METHODS

The Caris CODE database (Comprehensive Oncology Database Explorer) contains tumor molecular profile data for 841 patients with solid tumors in version 1.0. It also contains demographic information about the patients, their drug treatments that they received before and after molecular profiling, and records of their clinical outcomes. There are 92 breast cancer patients recorded, and this breast cancer cohort was mined after web scraping the data from the Caris website, to determine if molecular characterization recommendations influenced drug selection by their physicians after the time of profiling, and if any molecular subsets had different outcomes. Table 1 describes the clinical characteristics of the patients in this breast cancer cohort. According to Caris, 33% of the samples were from metastatic samples; 50% of these metastatic breast samples were from the lymph nodes, and the rest were from other sites.

The amount of time that patients were monitored varied, as shown in Figure 1. On average patients’ treatment records were available for 1327 days after diagnosis (1342 for matched treatment patients and 131 for unmatched), and on average the time of monitoring after profiling was 583 days. The longest period of monitoring after tumor profiling (the patient represented on the furthest right of Figure 1) was 1317 days; this was 1407 days after diagnosis. The longest amount of time that records were available, i.e. after diagnosis up until the last contact day, was 9427 days.

The data were analysed independently of Caris. Patients were covered under 1 of 4 different protocols or exemptions, listed as follows. (1). The Caris Registry Protocol (TCREG-001-00-V2-1209) was approved by WIRB (WIRB Tracking #20092285) and has an NCT# of NCT02678754. (2). The Caris POA Prospective Repository (COE-001-0815) was approved by WIRB (WIRB Tracking #20162864) and has an NCT# of NCT03324841. (3). The Caris POA Retrospective Repository (COE-002-0116) was approved by WIRB (WIRB Tracking #20162657) and has an NCT# of NCT 00326499. (4). ION data is covered under an IRB exemption. All data are retrospective and have been de-identified prior to Caris receiving it and authors performing independent analyses.

## Abbreviations

AR: androgen receptor
HER2: human epidermal growth factor receptor 2
IHC: immunohistochemistry
ER: estrogen receptor
OS: overall survival
PR: progesterone receptor
